# How Novel Algorithms and Access to High Performance Computing Platforms are Enabling Scientific Progress in Atomic and Molecular Physics

**Published:** 2015

**Authors:** Barry I. Schneider

**Affiliations:** Applied and Computational Mathematics Division National Institute of Standards and Technology* Gaithersburg, MD 20899-8910, USA

## Abstract

Over the past 40 years there has been remarkable progress in the quantitative treatment of complex many-body problems in atomic and molecular physics (AMP). This has happened as a consequence of the development of new and powerful numerical methods, translating these algorithms into practical software and the associated evolution of powerful computing platforms ranging from desktops to high performance computational instruments capable of massively parallel computation. We are taking the opportunity afforded by this CCP2015 to review computational progress in scattering theory and the interaction of strong electromagnetic fields with atomic and molecular systems from the early 1960’s until the present time to show how these advances have revealed a remarkable array of interesting and in many cases unexpected features. The article is by no means complete and certainly reflects the views and experiences of the author.

## 1. Introduction

The time independent and time dependent many-body Schrodinger equations show a remarkable array of features which often only emerge subsequent to laborious and time consuming calculations. Progress in the field has been marked by the development of novel theoretical and numerical methods, backed by solid algorithms, software development and the continuing evolution of computing platforms ranging from powerful desktops to supercomputers. In this article, I outline historical progress in the treatment of atomic and molecular continua in scattering processes and how these ideas have been extended to the time-dependent interaction of short, intense laser pulses with such targets.

### 1.1. Early History: The 1960’s and 1970’s

I begin with a short history of early developments in electron-atom/molecule collisions.

**Figure 1:**
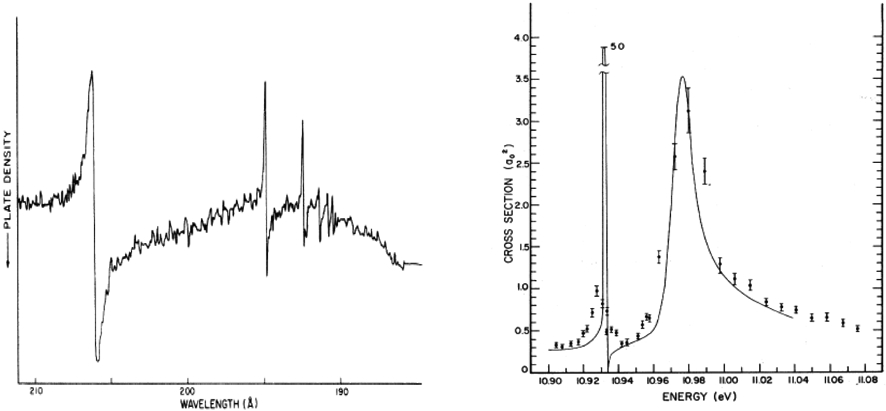
Some simple examples of resonances in atoms

- Computers advance sufficiently that quite high quality calculations could be performed on the scattering of electrons and photoionization of simple atoms such as H, He and Ne.
- Significant experimental advances; A number of experimental groups in AMO physics develop instruments able to extract high quality total and differential electron scattering cross sections and photoabsorbtion cross sections ( See Figure 1 [1, 3])

Densitometer trace of the absorption spectrum of He. The absorption is enhanced on the low-frequency side and reduced on the high-frequency side. See [1] Photoabsorption of H^−^. Comparison of theory (solid curve) [See [2]] and experiment (with error bars) [See [3]]. Data from experiment normalized to theory at 10.90 eV.

(iii) Computation (See 3.1 for discussion of close-coupling (CC)) and experiment discover interesting and in some cases unexpected features such as Feshbach and shape resonances in an otherwise smooth background continuum [4, 5]. Such long-lived, quasi-bound states were seen in nuclear scattering earlier.
(iv) Extension of CC to a number of first row atoms
(v) An intuitive but useful approach called the Stabilization method [6] developed to treat resonances ( 3.2 )
(vi) Recognition that better numerical approaches were needed: Early developments include the R-and-J-matrix methods, ( 3.3, 3.4 ) Kohn and Schwinger variational methods ( 4.1 ), and complex rotation/scaling ( 3.5 )
(vii) Extensions to small diatomic molecules started. Effects of nuclear motion are discussed.

Some comments. The CC approach primarily used well established numerical techniques, although special care was required to deal with long range interactions. The use of matrix and variational techniques really enabled much more complex and sophisticated calculations. These matrix based methods have proven their mettle and are even today the methods of choice for most problems.

### 1.2. Later Developments; 1980-present

From the 1980’s to the mid 2000’s there were a number of important computational advancements.

- R-matrix and Kohn variational methods become reliable in predicting the cross sections of even moderately complex polyatomic molecules
- New and more efficient discretization techniques such as the Finite Element Discrete Variable Representation were developed (FEDVR) 4.2
- Scattering methods were extended to the time-dependent domain where new experimental facilities such as free electron lasers are becoming available to study the interactions of short, intense laser radiation with atomic and molecular targets. Now possible to “see the electrons dance" and even control their motion using optical techniques. Looking directly at electron correlation effects becomes a reality computationally and experimentally

## 2. Personal View

In my opinion, the most effective approaches to treat problems in atomic and molecular systems involving a continuous spectrum, have been based on techniques which try to avoid the details of the asymptotic form of the wavefunction. There are a few options. One may divide either physical or function space into two ( or perhaps more ) regions. In the innermost region, one needs to deal with the full interacting set of nuclei and electrons but does not have to worry about the details of asymptopia. This inner region is then quite similar to a bound state problem and it becomes unsurprising that the most successful computational approaches to scattering borrow liberally from those developed by the atomic structure and quantum chemistry communities for discrete states. In the other regions, the Hamilitonian often "simplifies" in some way that makes it computationally easier to solve. In real space, one might be able to set the interaction to zero or to some problem where an analytic solution is available 3.3. In function space, one could assume the expansion coefficients of the asymptotic functions are known outside the interaction region 3.4. How the different regions are joined, and how the scattering information is extracted becomes the central difference of all the various approaches. There is also the possibility of avoiding the asymptotic region entirely by analytically continuing from the complex plane, rotating the scattering coordinates into the complex plane, or relating a discrete basis set expansion to a numerical quadrature. Among the first class of methods are the R-and-J matrix method, and the Kohn and Schwinger variational principles. The latter category is exemplified by either full or exterior complex scaling and the equivalent quadrature technique. Lets us look briefly at a few of these.

## 3. The Early Days; 1960-1980

### 3.1. Close Coupling

The close-coupling method [5] proceeds in the most straightforward manner; expand the scattering wavefunction in a set of target states times unknown one-electron scattering functions, insert into the Schrodinger equation, project onto the target states to obtain a set of coupled integro-differential equations and solve those equations by numerical integration. The close coupling or expansion in target states approach became routinely employed over the 1960’s-1970’s to both neutral and ionized species. The computed cross sections were used in astrophysical and fusion modeling. Computing photoionization cross sections became a simple matter of calculating the dipole matrix element between the electron ion wavefunction and the initial bound state, recognizing the dipole selections rules, a nice benefit once the scattering calculation has been performed. The method was extended to include pseudostates to account for the polarizability of the true target states and to represent the ionization continuum. However, it also became evident that the direct solution of the integrodifferential equations of scattering theory, was not the most efficient way to attack the problem numerically and other approaches were initiated.

R-Matrix Equations

(1a) $$\Psi_{k}=\sum_{c=1}^{n}\sum_{i=1}^{m}\psi_{c}u_{ci,k}^{0}a_{ci,k}+\sum_{q=1}^{M}\chi_{q}b_{q.k}$$

(1b) $$\left[H_{N+1}(r;R)+L_{N+1}-E(R)]\Psi (r;R)=\;\;L_{N+1}\Psi (r;R\right)$$

(1c) $$[H_{N+1}+L_{N+1}-E_{k}(R)]\Psi_{k}=0$$

(1d) $$L_{N+1}=\sum_{i=1}^{N+1}\frac{1}{2}\delta (r_{i}-a)\left(\frac{\partial }{\partial r_{i}}-b\right)$$

(2a) $$\;u_{c}(a)=(\psi_{c}|\Psi {)}_{r=a}=\;\sum_{d=1}^{n}R_{c,d}[\frac{\partial u_{d}(r)}{\partial r}-bu_{d}(r){]}_{r=a}$$

(2b) $$R_{c,d}=\frac{1}{2}\sum_{k}\frac{u_{c,k}(a)u_{d,k}(a)}{\left[E_{k}(R)-E(R)\right]}$$

(2c) $$u_{c,k}(a)=\sum_{i}a_{ci,k}u_{ci,k}^{0}(a)$$

### 3.2. Stabilization Method

**Figure 2:**
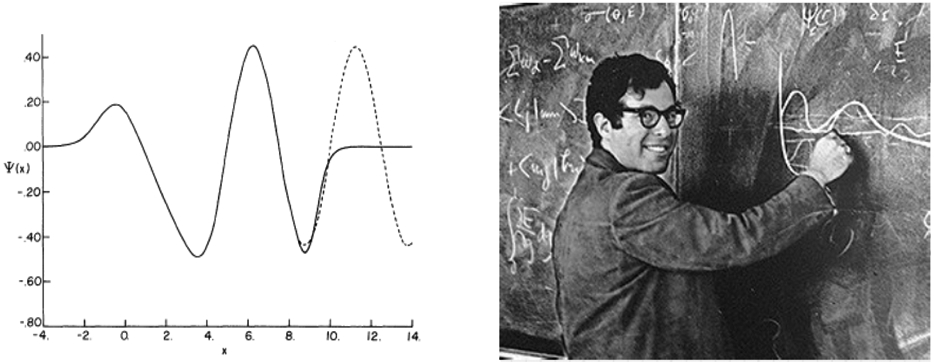
( (L) Comparison of exact and stabilization wavefunction in nonresonant region for simple model potential [6]. (R) Mentor and close friend Howard S. Taylor [9/17/1935-4/17/2015] circa 1970’s

The "Stabilization Method", was developed by A. Hazi and H. Taylor [6] ( See Figure 2 ) at USC. The success of the approach had a significant influence on much of the work that followed. Taylor correctly and intuitively argued that a discrete basis set expansion would converge, inside the range of the basis, to the true scattering function within an unknown normalization constant. By diagonalizing the Hamiltonian in a larger and larger discrete basis, certain eigenvalues "stabilized" with basis set size. Those eigenvalues that stabilized, were the resonances, and they stabilized simply due to the fact that resonant wavefunctions tend to be localized with rather small asymptotic tails. It was also noticed that the basis could describe the background continuum, but again only to an unknown normalization factor. There are a number of approaches which utilized the stabilized solution to find estimates of the width. Some are motivated by the connection between the stabilized eigenfunction and the variational method proposed by Harris [7]. Another method used the diagonalization to find an approximation to the background continuum function and then computed the width via the Fermi Golden Rule and an approximation to the density of states suggested by Miller [8].

### 3.3. The R-Matrix Method

**Figure 3:**
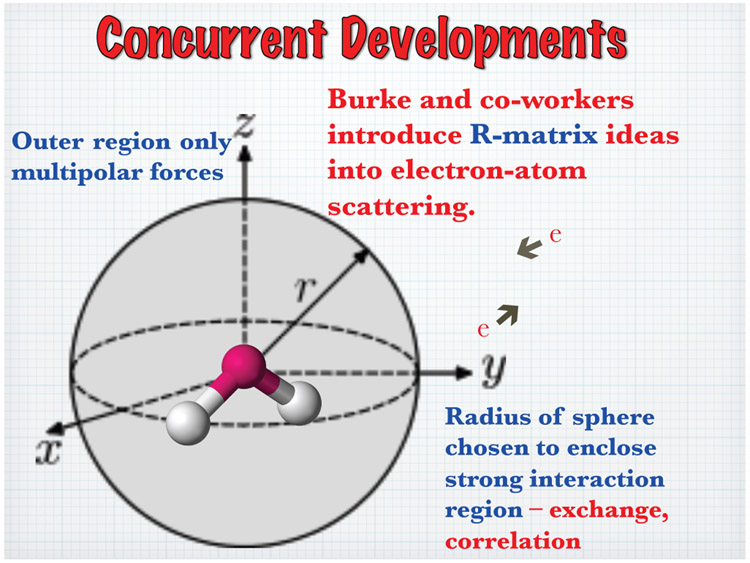
Partitioning Space in the R-Matrix Method into an Inner and Outer Region

**Figure 4:**
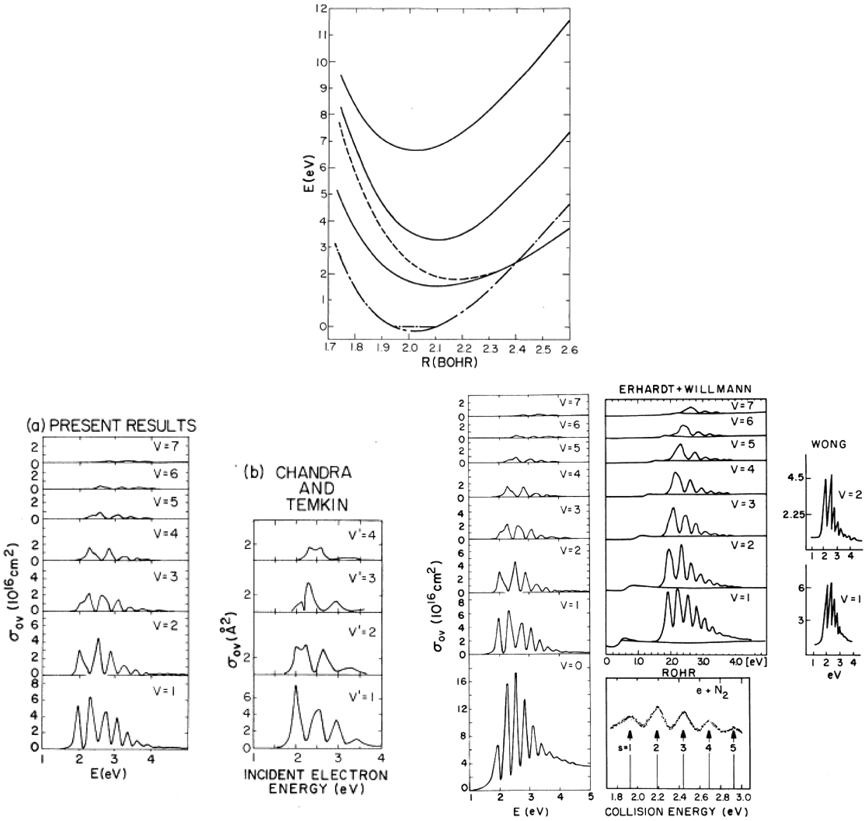
Upper Panel: $N_{2}^{-}$ R-matrix Born-Oppenheimer Potential Curves. Lower Panels: Comparison of Vibrational Excitation Cross Sections. (L) R-Matrix [14] vs Vibrational Close-Coupling [15] and (R) Theory [14] vs Experiment. [16, 17].

The R-matrix method was developed in nuclear physics by Wigner and Eisenbud [9]. Phil Burke [10] and his colleagues in the UK, turned the R-matrix method into one of the most successful computational approaches to treat the scattering of electrons from atoms. A few years later, Schneider [11, 12] generalized the method to treat electron scattering from molecules and in a significant development [13, 14] showed how the theory could be used effectively to treat nuclear motion beyond the usual adiabatic approximation. The central R-matrix idea, including the relevant equations, is shown in Figure 3. One encloses the strong interaction region in a sphere, imposes a boundary condition on the surface of the sphere to get a discrete eigenvalue problem, solves that problem for its eigenvalues and eigenfunctions and uses that information to extract the scattering matrix by matching to asymptotically known functions. In contrast to nuclei, where one may assume that the potential is zero outside the interaction region, the atomic scattering problem needs to treat the long-range multipolar interaction of the electron and target more precisely. In the atomic R-matrix method this is done using solutions to coupled long range *differential* equations using these multipolar potentials. One may also take the R-matrix on the inner sphere and directly propagate it to larger distances using the coupled differential equations. The external region is devoid of exchange and electron correlation and this makes for a much simplified treatment. The functions used to expand Ψ_*k*_ are completely arbitrary. In practice, they are chosen such that the *ϰ_q_* vanishes at *r* = *a*. The channel functions, *ψ_c_*, describe all the internal coordinates of the residual target and $u_{ci,k}^{0}$ is a radial basis function which is non vanishing at *r* = *a*. The $u_{ci,k}^{0}$ contain all the information needed for extracting the scattering information in channel *c*. The unknown coefficients *a* and *b* are determined by inserting the expansion of the wavefunction (1a) into (1c), projecting onto the basis states, thus reducing the equation to the diagonalization of a large matrix. Once this diagonalization has been performed, the R-matrix and therefore the scattering information may be calculated at any energy by matching at *r* = *a*, a very efficient process. At this point we have fixed the nuclei in space. This restriction may be relaxed [13, 14] to include nuclear motion. One approach which works away from threshold and away from resonances, is to take the fixed nuclei scattering amplitudes at a series of geometries, multiply by a ro-vibrational wavefunction and integrate over the nuclear coordinates. Near a resonance this does not work since the electron is trapped in a quasi-bound state and the molecular cloud wants to adjust to the change in the field but they do so adiabatically. So, the essence of the Born-Oppenheimer approximation is retained. The compound state vibrational energies determine the positions of the resonances and the overlap of the compound state and neutral vibrational eigenfunctions (Franck-Condon factors ) are the strengths. The lower left panel in Figure 4 compares the vibrational excitation cross sections from [15] and [14]. Note that [15] fails to reproduce the details of the compound levels. The lower right panel compares [14] with experiment [16, 17]. The calculations agree with experiment quite well and in addition, allowed the absolute normalization of the cross sections to be determined for the first time. Finally, I must mention a new and extremely robust R-matrix approach, using a B-spline, non-orthogonal basis, that has been developed and applied to electron atom collisions by Zatsarinny [18]

### 3.4. The J-Matrix Method

The J-Matrix method was developed by Reinhardt, Heller, Yamani and Fishman [19, 20, 21]. Applications to both single channel and multichannel problems may be found in the literature. [22, 2]. The basic idea is straightforward. For many simple potentials, for example, the free particle or coulomb potential, there are tri-diagonal, analytic expressions available for matrix elements of the appropriate one-particle Hamiltonian in a known *L*^2^ basis. There are also analytic expressions for the coefficients of the regular and irregular solutions to these one particle operators which are exploited in the final solution of the scattering problem. The fundamental idea is quite similar to the R-matrix theory. The matching in space to the unknown linear combination of regular and irregular solutions to the asymptotic Hamiltonian is replaced by the analytically known expansion coefficients when the potential has assumed its asymptotic form. The coefficient joining the two solutions provides the scattering information. All the unknown coefficients are determined by projection onto the complete basis set using the known tridiagonal form of the asymptotic Hamiltonian in the basis.

### 3.5. Complex Scaling

**Figure 5:**
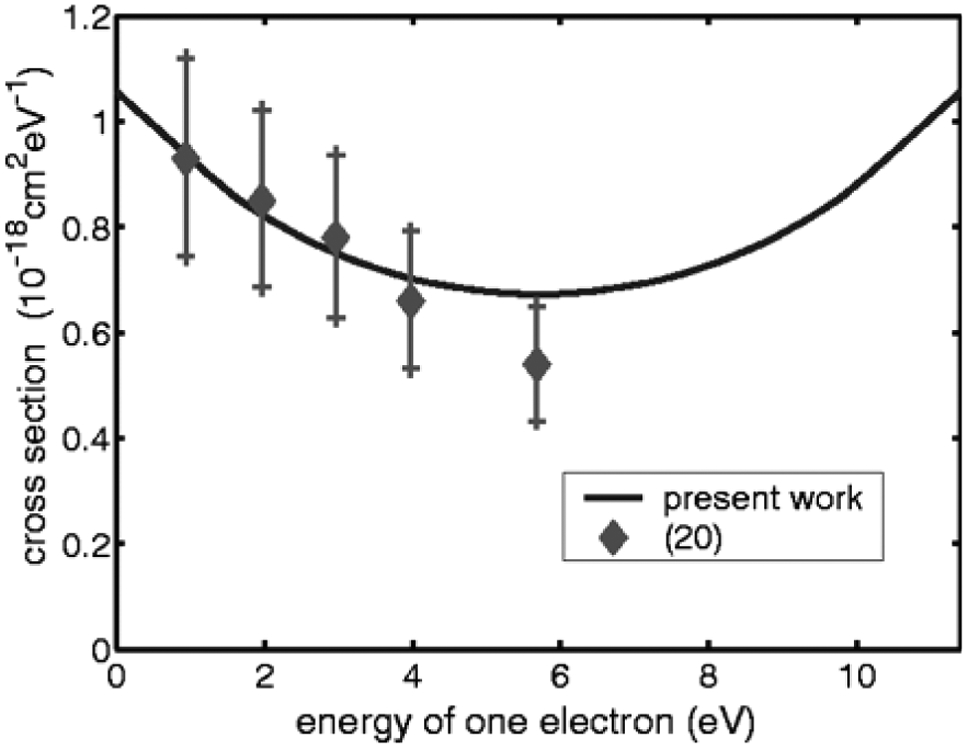
Single Differential ionization cross section for e^−^ + H collisions at 25eV incident energy. At 5.7eV there is equal energy sharing as expected.

**Figure 6:**
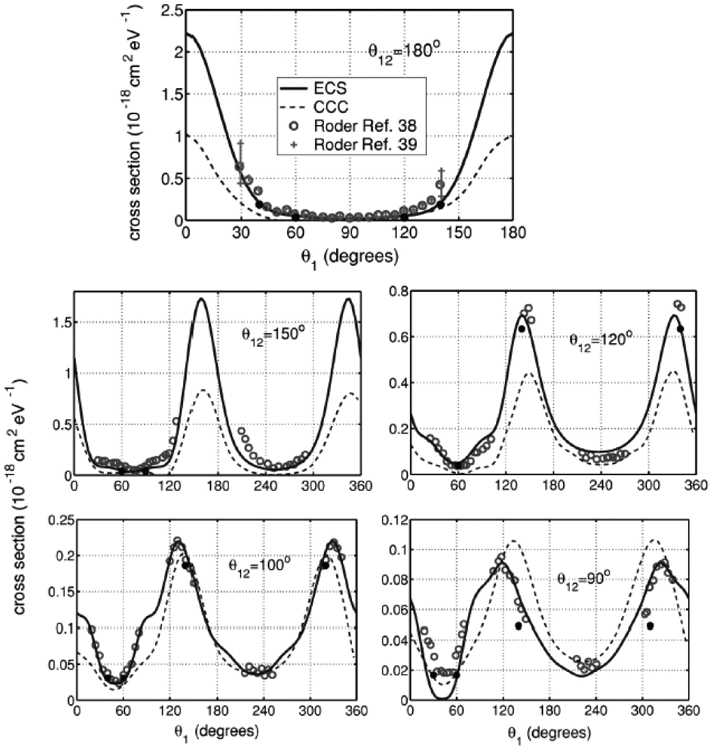
Coplanar triple differential cross section for 17.6-eV incident e^−^ + H collisions. ECS(solid), Convergent Close Coupling (broken) and experiment(dots). Experimental cross sections are absolute. See [28]

Simon [23, 24] extended and made the work of Balslev and Combes [25] on the spectral theory of Schrödinger operators known to atomic physicists. These authors introduced the original concept of complex scaling (let *r* → *r* exp(*iθ* ), an approach that was quickly exploited by a number of researchers, particularly the quantum chemistry community, as a practical tool to compute the positions and widths of scattering resonances. An unfortunate problem was encountered early on in that the complex scaling made the computations of the integrals involving the now, highly oscillatory inner orbitals, quite difficult. Another issue, raised by McCurdy and Rescigno [26] was that complete complex scaling would not work for molecules in a practical sense ( within the BO approximation ) and suggested the use of a mixed set of real and complex basis functions as a substitute. Simon [27] put the idea of using exterior complex scaling (ECS) on a firm mathematical foundation and this method remains one of most robust approaches even today. It has been advantageously used to produce the first truly accurate calculation of impact ionization of H [28] (Figures 5 and 6 )

### 3.6. Equivalent Quadrature

The idea of relating the diagonalization of a Hamiltonian in an *L*^2^ basis to a numerical quadrature was put forward by Heller, Reinhardt and Yamani [29]. This relationship yields a numerical approximation to the Greens’s function which properly treats the i*ε* limit required in the scattering problem. The concept was interesting from a historical perspective but never took off as a consequence of the difficulty of having to relate the *L*^2^ basis to a quadrature. This can be done only for a few basis sets. I mention it here for completeness.

## 4. The Modern Era;1980-Today

### 4.1. The Kohn Variational Methods

**Figure 7:**
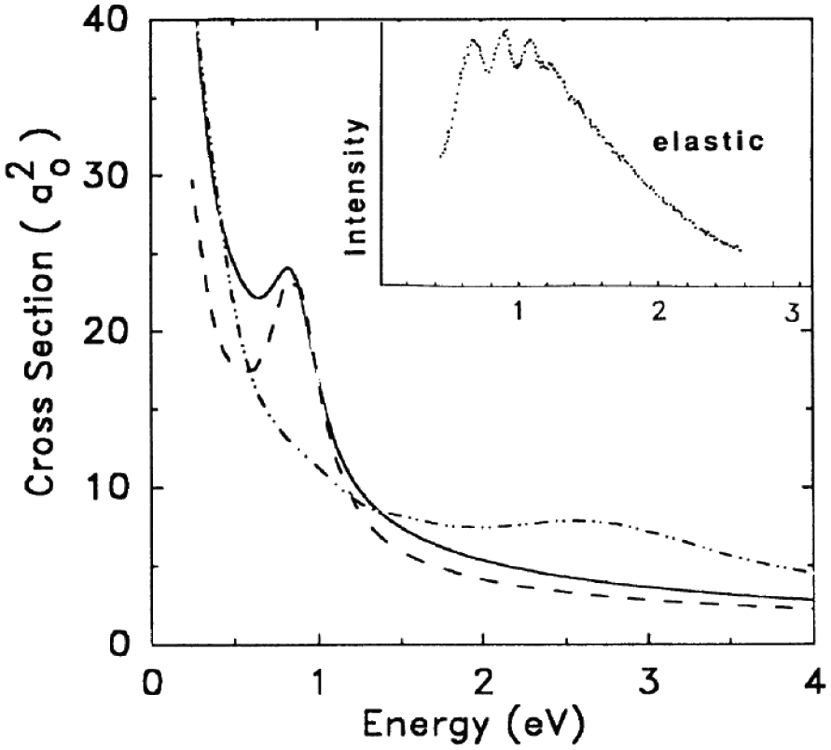
e + CH_2_O exhibiting a π* antibonding shape resonance.[37]

**Figure 8:**
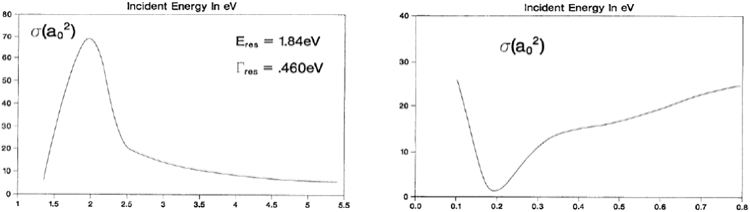
e + C_2_H_4_ scattering. (L) Exhibiting a π* antibonding shape resonance and (R) a ^2^A_*g*_ Ramsauer-Townsend minimum. [38]

Arguably, the Kohn [30, 31, 32, 33, 34] (KVP) and the Schwinger [35, 36](SVP) variational methods, have, along with the R-matrix method, been the most productive of the methods developed for the electron scattering problem. The Schwinger variational method has the advantage that it does not require the trial function to have the correct asymptotic behavior. A practical disadvantage is that it is based on an integral equation, and requires matrix elements of the Green’s function. Here I will concentrate on the Kohn variational method. The Kohn stationary principle is stated in Equation 3 and the trial wavefunction in Equation 4. Depending on the choice of the long range functions, *R* and *I*, *O* can be the *T*, *S* or *K* matrix. The trial wavefunction is inserted into the Schrodinger equation and then projected onto the basis to get a large set of linear equations for *d* and *O*. The first two summations in (4) contain only *L*^2^ functions and are chosen to account for all of the details of electron correlation and exchange as well as the inner parts of the scattering functions. *R* and *I* are regular at *r* = 0, are chosen to be orthogonal to *ψ_q_* and to go asymptotically to the regular and irregular solutions of an appropriately chosen model problem, such as a free wave or a coulomb function. The terms on the right hand side of the linear equations involve matrix elements containing the known functions, Φ_Γ_*R*_Γ_. If we define the projection operators as in Equation 5 then one may formally eliminate the terms involving the Qspace functions in terms of an energy dependent, non-local, optical potential, where *P* now contains only the functions, Ξ_Γ_*ψ_q_*, and Φ_Γ_*I*_Γ_. Formally solving the linear equations for the trial value of *O* and then substituting its value back into the Kohn expression, gives the final stationary value of [**O**], Equation 6. **M** consists of matrix elements of the optical potential between the functions Ξ_Γ_*ψ_q_*, Φ_Γ_*R*_Γ_ and Φ_Γ_*I*_Γ_. Importantly, forcing *R* and *I* to be orthogonal to *ψ_q_* eliminates any difficult free-free and bound-free matrix elements from the calculation. Only overlap integrals of these functions are required. If the KVP is used with principal value boundary conditions, the **M_qq_** can be zero leading to what are known as the Kohn anomalies. While there are various ways to get around this problem, it turns out that when outgoing wave boundary conditions are imposed, the *M_qq_* matrix is complex symmetric and at real energies there are no anomalies. This approach termed the Complex Kohn Variational Method is therefore preferred albeit one must deal with complex rather than real matrices. Two examples of the application of the complex Kohn method to polyatomic molecules are shown in Figures 7 and 8 [37, 38].

Kohn Variational Principle

(3) $$[O_{\Gamma ,\Gamma^{\prime }}]=O_{\Gamma ,\Gamma^{\prime }}-2\langle \Psi^{\Gamma }|H-E|\Psi^{\Gamma^{\prime }}\rangle$$

Trial Wavefunction

(4) $$|\Psi^{\Gamma }>=\sum_{\Gamma^{\prime },q}d_{\Gamma^{\prime }q}^{\Gamma }|\Xi_{\Gamma^{\prime }}(X_{N})\psi_{q}(X_{N+1})>\;\;+\sum_{K}d_{K}^{\Gamma }|\Theta_{K}(X_{N+1})>\;\;+\sum_{\Gamma^{\prime }}|\Phi_{\Gamma^{\prime }}(X_{N},\Omega_{N+1}>\left[R_{\Gamma^{\prime }}^{\Gamma }\delta_{\Gamma \Gamma^{\prime }}+O_{\Gamma ,\Gamma^{\prime }}I_{\Gamma^{\prime }}^{\Gamma }\right]$$

Projection Operators

(5) $$Q=\sum_{\mu }|\Theta_{k}><\Theta_{k}|\;P=I-Q$$

The Effective Matrix Hamiltonian

$$\;H_{\mathrm{eff}}=\;H_{\mathrm{PP}}+(H-E{)}_{\mathrm{PQ}}(E-H{)}_{\mathrm{QQ}}^{-1}(H-E{)}_{\mathrm{QP}}$$

The Stationary Value of [**O**]

(6) $$\left[O]=-2(M_{00}-M_{0q}M_{qq}^{-1}M_{q0}\right)$$

### 4.2. Time-Dependent (TD) Processes

**Figure 10:**
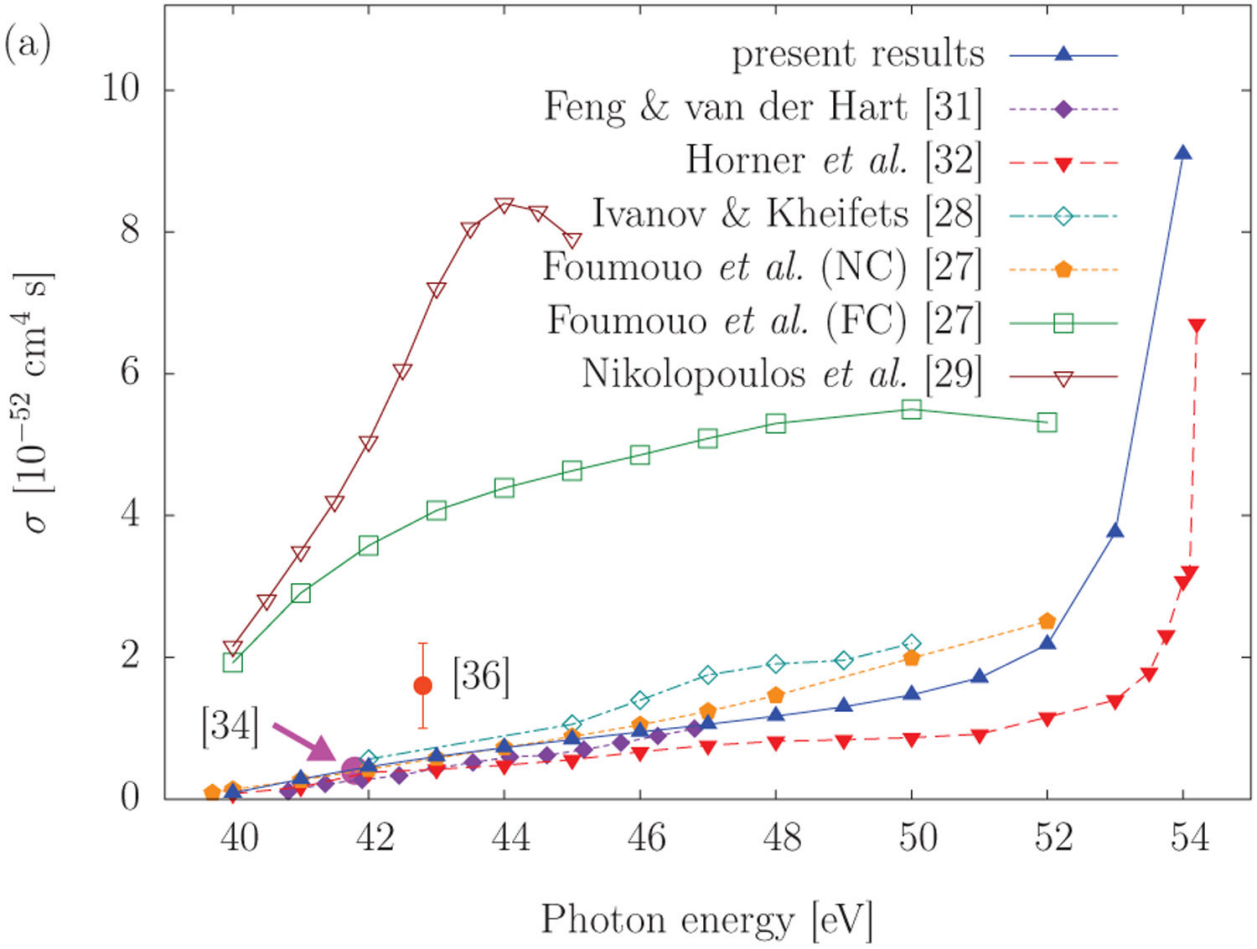
Comparison of a number of theoretical calculation of the direct double photoionization of He [See [40]]. Also shown are two experimental measurements.

**Figure 11:**
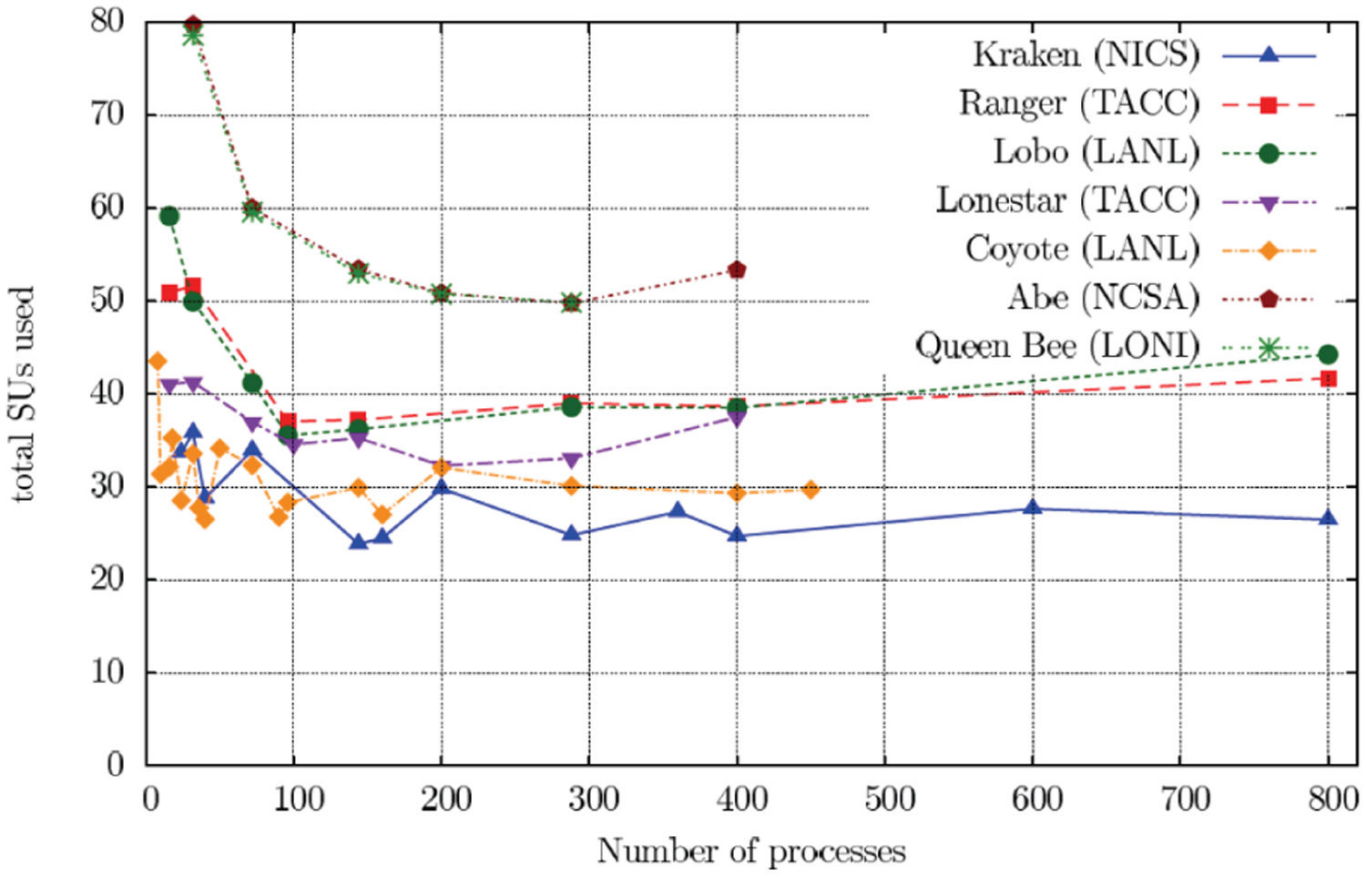
Scaling of FEDVR/SIL on Supercomputers. Plotted are total service units (≈ core hours) on the x-axis. and processor number on the y-axis. Strong scaling would be characterized by a perfectly flat curve.

In the past ten years there has been a significant effort devoted to computational studies of the interaction of intense, short pulse (attosecond) electromagnetic fields with atoms and molecules. The computational effort that is required is substantial, even for relatively small atomic and molecular targets. As a consequence, it is not surprising that the use of supercomputers and parallel processing techniques have become the norm. In the TD approach, one works with wavepackets [ having an energy spread ], needs to [ often ] propagate for long distances and times and then extracts the scattering information by projection onto asymptotic states. The plus side is that there is no need to impose boundary conditions on the wavefunction, which can be difficult to accomplish in ionizing collisions. Space limitations prevent only a brief look at this fascinating and growing field of physics. Arguably, the most effective approach to the space discretization in one and two-electron atomic and molecular systems is the FEDVR [39]. The advantage of this approach in practical calculations is that it leads to a sparse and structured Hamiltonian matrix which is amenable to efficient parallelization. The FEDVR discretization procedure is briefly shown in Equation (7). When coupled to the Short Iterative Lanczos (SIL) process as outlined in Equations (8), (9) and (10), one obtains a powerful computational approach to the TD Schrödinger equation. As can be seen in Figure 10, the method has been successfully applied to the double ionization of the He atom from the energy region where only direct double ionization is possible up to the sequential threshold. The scaling of the method on a variety of parallel processors is shown in Figure 11. One should note that after an initial erratic beginning, the curve flattens indicating that each core is now performing 1/*n* of the total work. As one can see, only very sophisticated numerical methods are able to reproduce the experimental results. The large rise of the cross section approaching the sequential threshold has been called virtual sequential double ionization in analogy to what is seen from the pole structure due to a resonance. There are two important points to emphasize. The first is that for many problems only very refined physical models, requiring intensive computation, can be relied upon to be predictive. The second is that it is critical to develop algorithms and software that scale well with processor count. A nice summary of what has been happening in the area of attosecond physics may be found in [41]

Summary of FEDVR Discretization

- Within each finite element:
  (7a) $$u_{i}(x)=w_{i}\sum_{n}\phi_{n}(x)\phi_{n}(x_{i})$$
  (7b) $$u_{i}(x_{j})=\delta_{i,j}$$
  (7c) $$\langle u_{i}|x|u_{j}\rangle =x_{i}\delta_{i,j}$$
  (7d) $$\langle u_{i}|F(x)|u_{j}\rangle =F(x_{i})\delta_{i,j}$$
  where *ϕ_i_*(*x*) are members of a classical set of orthogonal functions and *x_i_* and *w_i_* are the corresponding Gauss quadrature points and weights.
- Connection between elements
  The elements are connected by ensuring that the functions at the element boundaries are continuous.

The structure of the Hamiltonian in one dimension is,

Summary of TD Propagation

Short Time Lanczos (SIL) propagator

(8) U(t+δt)≈exp[−iH(t)δt]

The Three Term Lanczos Recursion

(9a) $$\beta_{n+1}|n+1\rangle =|q\rangle =[H(t)-\alpha_{n}]|n\rangle -\beta_{n}|n-1\rangle$$

(9b) $$\alpha_{n}=\langle n|H(t)|n\rangle$$

(9c) $$\beta_{n+1}=\langle q|q\rangle$$

The Exponential Propagator in the Diagonal Basis

(10) $$\langle a|\;\text{exp}[-iH(t)\delta t]|C\rangle =\;\;\sum_{n}\langle A|n\rangle \;\text{exp}[-i\lambda_{n}\delta t]\langle n|B\rangle$$

## 5. Conclusion

In this article I have tried to give the reader a flavor of many of the exciting theoretical and computational developments in atomic and molecular physics since it became possible to perform large scale calculations on computers. The early pioneers has to contend with slow processors and tiny memories by todays standards. Luckily, their processors and memories were quite sophisticated. It is a tribute to them that even with substantial digital obstacles they were able to produce many new and interesting results. Today, we are looking towards computers that will operate at the exascale level. The challenges to use that computer power effectively are formidable. If we can meet these challenges by developing the algorithms and software to use these new devices, I am sure the rewards will be significant and we will the truly be able to control atoms and molecules at their most fundamental level.

## Figures and Tables

**Figure 9:**
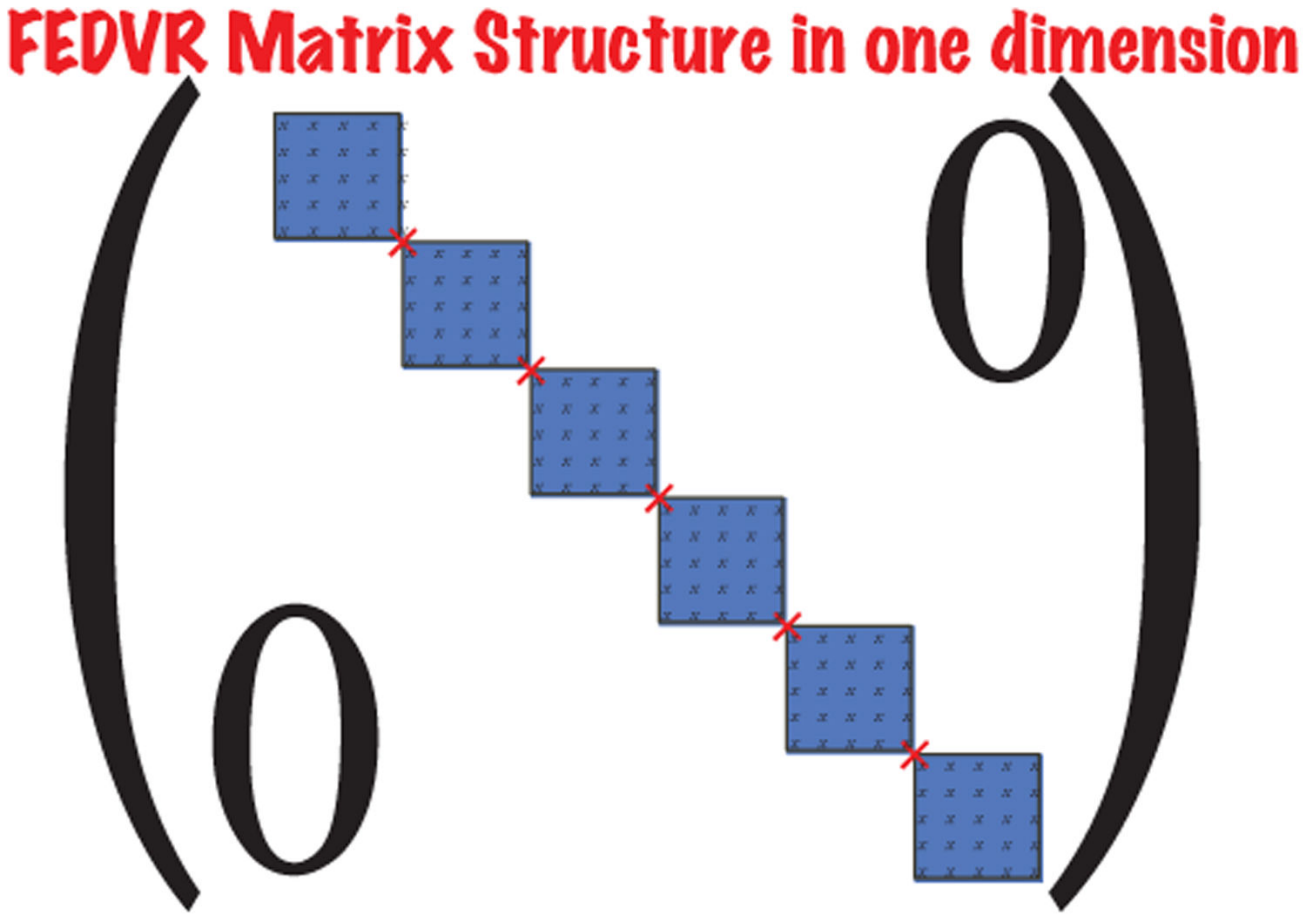
FEDVR Hamiltonian in 1D

**Table 1:** Performance of the He FEDVR code on various platforms.

Name	CPU’s	GHz	cores/CPU	CPU’s/cores per node
Coyote	Opteron	2.60	1	2/2
Lobo	Opteron	2.20	4	4/16
Ranger	Opteron	2.30	4	4/16
Lonestar	Xeon	2.66	2	2/4
Abe	Xeon	2.33	4	2/8
Queen Bee	Xeon	2.33	4	2/8
Kraken	Opteron	2.33	4	2/8
